# K36-based inhibitor analogs as potential therapeutics against SARS-CoV-2 main protease (Mpro): a computational investigation

**DOI:** 10.1038/s41598-025-06676-5

**Published:** 2025-06-23

**Authors:** Mohamed L. AbouYoussef, Mohamed M. Aboelnga, Nemany A Hanafy, Khaled E. El-Kelany

**Affiliations:** 1 Institute of Nanoscience and Nanotechnology, Kafrelshiekh University, Kafrelskiekh, 33516 Egypt; 2 Chemistry Department, Faculty of Science, Damietta University, New Damietta, 34511 Egypt; 3 Faculty of Science, King Salman International University, Ras Sudr, 46612 Sinai Egypt

**Keywords:** Computational biology and bioinformatics, Drug discovery, Chemistry

## Abstract

The global pandemic caused by SARS-CoV-2 has underscored the critical necessity for effective antiviral therapies. The viral main protease (Mpro), crucial for viral replication, has emerged as a promising therapeutic target. In the present study, the inhibitory potential of ten drug-like compounds (KL1-KL10), designed as derivatives of the parent inhibitor K36, against Mpro, has been computationally investigated. To elucidate the binding affinities and interactions of the suggested drugs with the Mpro active site, molecular docking and molecular dynamics (MD) simulations till 500 nanoseconds have been applied. Our results revealed that many suggested inhibitors exhibited enhanced binding affinities compared to the parent inhibitor K36. Among these, KL7 displayed the most favourable binding characteristics, with a docking score of -13.54 and MM-PBSA binding energy of -34.57 kJ/mol, surpassing that of K36. Molecular dynamics simulations demonstrated persistent binding of these compounds to Mpro, with RMSD values ranging from 0.5 to 2.0 nm, suggesting their potential as effective inhibitors. These findings suggest that the proposed ligands hold promise as potential scaffolds for developing potent antiviral drugs against COVID-19.

## Introduction

The COVID-19 pandemic, caused by the severe acute respiratory syndrome coronavirus 2 (SARS-CoV-2), highlighted the crucial need for improved antiviral therapies. While vaccinations have played a critical role in minimizing the pandemic’s impact, the ongoing appearance of novel strains and the possibility of diminishing immunity underline the significance of developing antiviral medications as a backup option. As of July 2024, global COVID-19 cases have reached $\sim$775 million with $\sim$7 million deaths^1^, while the dominant JN.1 subvariants (KP.2/KP.3) account for $\sim$85% of sequenced cases^2^. Hospitalizations and deaths remain lower than pandemic peaks, though seasonal surges persist, with the U.S. reporting $\sim$300 weekly deaths^2^ and Europe noting winter 2023-24 case increases^3^. Approximately 70% of the global population has received at least one vaccine dose^4^, significantly reducing severe outcomes despite ongoing challenges like Long COVID (affecting 5–20% of survivors). For real-time updates, one can find more data elsewhere^1–3^. Among the variety of possible therapeutic targets in the viral proteome, the main protease (Mpro, also known as 3CLpro, PBD ID: 6WTJ) has emerged as a primary target due to its critical role in viral replication and its differences from human proteases^5–8^.

Mpro is a cysteine protease that plays an important role in the viral life cycle, responsible for the proteolytic processing of the viral polyproteins pp1a and pp1b. These polyproteins serve as precursors for a variety of non-structural proteins (NSPs) required for viral replication, such as RNA-dependent, RNA polymerase, helicase, and other viral assembly enzymes^9,10^. Mpro cleaves these polyproteins at eleven particular locations, which is required for the production of functional proteins and the conclusion of the viral life cycle. As a result, inhibiting Mpro can effectively stop viral replication, making it a promising target for antiviral medication research. Moreover, a key advantage of targeting Mpro is its evolutionary conservation among coronaviruses. Although coronaviruses exhibit significant genetic diversity, Mpro retains remarkable sequence and structural homology across strains^11,12^.

Furthermore, Mpro has a distinct advantage as a therapeutic target due to structural and functional differences with human proteases^13,14^. Mpro’s active site features a catalytic dyad of Cys145 and His41, which distinguishes it from the majority of human proteases. This dissimilarity minimizes the possibility of off-target effects, potentially improving the safety and efficacy of Mpro inhibitors in clinical settings.

Mpro structure is extremely conserved across coronaviruses, with two identical protomers forming a homodimer. Each protomer consists of three domains: domains I and II create a chymotrypsin-like fold that contains the active site, while domain III is involved in dimerization and substrate recognition^15,16^. Mpro’s substrate-binding pocket is relatively shallow and solvent-exposed, making it difficult to create small-molecule inhibitors with high binding affinity and specificity. However, the presence of a conserved cysteine residue (Cys145) near the active site allows for the development of covalent inhibitors, which can form irreversible or reversible bonds with the enzyme, resulting in powerful and long-lasting suppression.

Previous studies^17,18^, have identified K36 as a possible covalent inhibitor of Mpro. This K36 compound binds to the catalytic Cys145 residue in the active site, establishing a persistent thiohemiacetal link that permanently inhibits the enzyme. While K36 has significant antiviral activity against SARS-CoV-2, its therapeutic use is limited due to low oral bioavailability and probable off-target effects^19^. As a result, the development of K36 analogs with enhanced pharmacokinetic and pharmacodynamic properties is critical for expanding its therapeutic applications. The selection of K36 (the *S*-form stereoisomer of GC376) as our parent compound is supported by its well-documented role as a covalent inhibitor of SARS-CoV-2 Mpro, as demonstrated by crystallographic studies (PDB ID: 7CB7) and fragment molecular orbital (FMO) calculations^20^. Although K36 displays lower occupancy than its B1S stereoisomer, its binding mode remains particularly valuable for drug development, featuring: (1) a reactive carbonyl warhead that specifically targets Cys145, and (2) favorable interactions with key active site residues (Glu166, Gln189). This combination of features establishes K36 as an excellent scaffold for structural optimization. Our strategy focuses on leveraging this characterized framework while systematically addressing its limitations to develop next-generation inhibitors with enhanced potency and improved pharmacological properties.

COVID-19, caused by the SARS-CoV-2 virus, affects the body in multiple stages:^21–25^ **Entry:** When an infected individual coughs, sneezes, or speaks, respiratory droplets are released into the mouth, nose, or eyes, which is how the virus enters the body. **Binding:** The spike protein of the virus attaches itself to ACE2 receptors on the surface of respiratory tract cells, especially those in the lungs. **Entry into the Cell:** After attaching itself to a cell, the virus merges with the cell membrane to release its genetic material (RNA). **Replication:** To produce new viral proteins and RNA copies, the virus’s RNA takes control of the cell’s machinery. **Assembly and Release:** After the new viral components have come together to form new viruses, the cells release the infected viruses to infect other cells. **Immune Response:** After identifying the virus, the body’s immune system mounts an attack. This immune response may occasionally become dysregulated and harm the body’s own tissues. **Spread:** The virus has the ability to be transfered in to other bodily organs, such as the kidneys, brain, blood arteries, and heart, where it may cause more harm.

Experimental studies have been useful in determining the effectiveness and safety of several medicines for COVID-19 therapy. Remdesivir, an antiviral originally designed for Ebola, showed excellent results in suppressing virus multiplication in vitro and in vivo, prompting its emergency use permission^26^. Dexamethasone, a corticosteroid, demonstrated substantial mortality reduction in hospitalized patients requiring oxygen or mechanical breathing in randomized controlled studies, showing its anti-inflammatory properties^27^. Monoclonal antibodies that target the SARS-CoV-2 spike protein, such as bamlanivimab/etesevimab and casirivimab/imdevimab, have been produced and evaluated in clinical studies, demonstrating their capacity to neutralize the virus and block cell entrance^28^. Additionally, convalescent plasma treatment and antiviral protease inhibitors have been investigated, albeit with different degrees of effectiveness and ongoing study^29,30^. While vaccines mitigate SARS-CoV-2 severity, viral evolution demands complementary therapies like Mpro inhibitors (e.g., Paxlovid, ensitrelvir)^31–33^. Future drugs must optimize oral bioavailability and eliminate ritonavir dependence to avoid metabolic interference.

Computational studies^34–38^ have significantly accelerated COVID-19 drug discovery and development. Drug repurposing initiatives have used virtual screening and molecular docking to discover current medicines that have potential antiviral action against SARS-CoV-2 targets such as the spike protein, Mpro^39^, and RdRp^40^. Structure-based drug design and molecular simulations have helped to develop new inhibitors for viral enzymes^7^. Furthermore, computational methods have helped to optimize antibody designs, forecast vaccine immunogenicity, and track viral evolution, all of which have contributed to the development of effective antiviral treatments^6,41–46^.

**Fig. 1:**
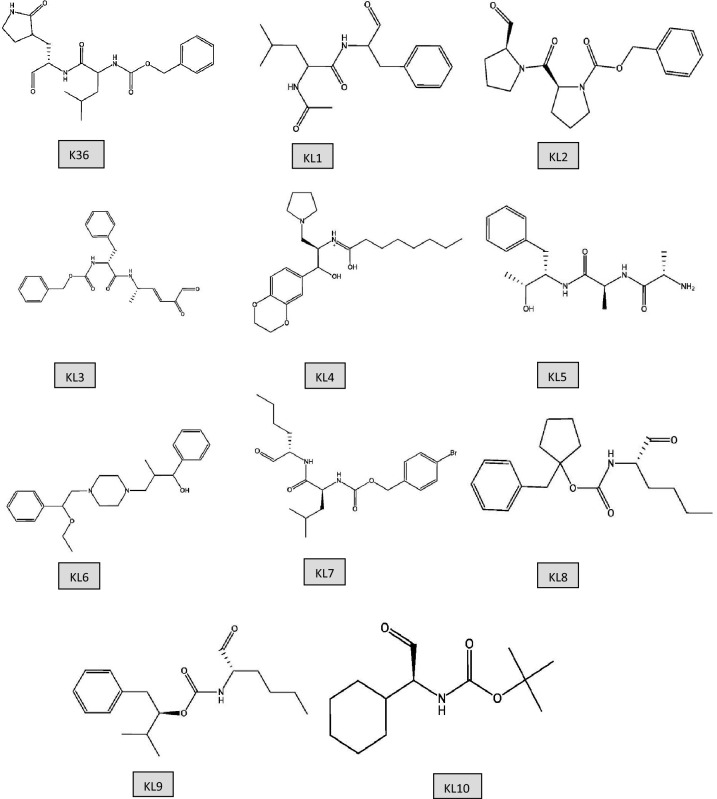
Chemical structure of the suggested ten K36-analogues inhibitors obtained using Swiss-Param similarity website. K36 refers to the experimental Mpro inhibitor, while KL1-10 represent the suggested ones.

In this investigation, we used a comprehensive computational method to suggest, assess, and test a series of K36-based analogs that might inhibit SARS-CoV-2 Mpro. The study included the following main steps: **Design of K36 analogs:** We created a wide library of K36 analogs, see Figure 1, by making structural changes to the scaffold while keeping the critical functional groups (-CHO and/or =CHOH) required for covalent binding to Mpro. The adjustments attempted to improve the analogs’ pharmacokinetic and pharmacodynamic features, such as solubility, permeability, and metabolic stability. This step was accomplished using the SwissParam website^47^ and resulted in the selection of ten similar items to K36 as shown in Figure 1, all of which were recorded and each inhibitor has its own drug bank number. **Molecular docking and MD simulations:** We used molecular docking and MD simulations to estimate the binding modes, affinities, and stability of the proposed analogs to Mpro^48^. These simulations gave important insights into the molecular basis of drug-target interactions, allowing us to select prospective lead compounds with high binding affinity and stability.

## Computatinal tools and methods

The study of molecular interactions and dynamics in biological systems frequently depends on computational methods that give insights beyond the scope of experimental procedures. Ten K36-analouges inhibitors (KL1 to KL10) chosen via using Swiss-Param similarity website^49^, based on the K36 core and its functional group, see Figure 1. All the studied ligands have its own drug bank number (DB), which recorded in Table 1.

**Table 1:** The Drug Bank ID for each chosen ligand recorded with its available traditional name.

Liagand	KL1	KL2	KL3	KL4	KL5	KL6	KL7	KL8	KL9	KL10
Drug Band ID	DB07749	DB03535	DB01810	DB09039	DB08038	DB08990	DB04234	DB07593	DB07592	DB04523
Trade name	-	Z-Pro-Prolinal	-	Eliglustat	-	Eprazinone	-	-	-	-

This study used standard, non-covalent, molecular docking and molecular dynamics (MD) simulations to examine the binding affinity and stability of the above mentioned ten (10) putative inhibitors of the SARS-CoV-2 Mpro. The non-covalent docking investigations were carried out with MOE 2009^50^, while the MD simulations were performed employing GROMACS 2021.3^51^. These computational tools made it easier to identify and characterize critical protein-ligand interactions, which helped to better understand inhibitor binding processes and guided the development of more effective Mpro inhibitors. This section describes the precise computational processes and settings used in the docking and MD simulations.

Molecular docking utilized to evaluate the ligand’s binding affinity and selectivity, as well as to identify important interactions involved in binding. Experimental studies^18^ in which the protein was extracted with the inhibitor show that the inhibitor binds to the protein by making a covalent bonding with Cys145. This type of covalent connection is relatively strong and persistent, allowing the inhibitor to effectively impede the function of the main protein.

MOE 2009.10 (Chemical Computing Group)^50^ was used to run molecular docking simulations on a local PC with an Intel Core i9-10900K CPU, 64 GB RAM, and the Ubuntu 20.04 operating system. The crystal structure of SARS-CoV-2’s main protease (Mpro) (PDB ID: 6WTJ)^6^ was prepared for docking by protonation with Protonate3D^52^, removal of crystallographic water molecules, and assignment of the docking site, which included the catalytic dyad residues His41 and Cys145. Energy optimization by using the MMFF94x force field were applied for the Mpro and ligands^53^. Partial charges were added using the AM1-BCC approach^54^. The MOE docking protocol employs the software’s internal representations for both receptor and ligand, with the binding site defined by ligand atom positions and incorporating receptor/solvent atoms within a 10 Å wall constraint. Using the Triangle Matcher algorithm for initial placement and rigid receptor refinement, the process evaluates interactions through combined London dG and GBVI/WSA dG scoring functions, generating the top 30 poses which are output to a dock.mdb file with molecular fingerprint generation enabled^55^.

Molecular dynamics (MD) simulations enhance molecular docking by offering a dynamic perspective of ligand-protein interactions. This technique simulates the movement of atoms and molecules over time, allowing for the evaluation of the ligand-protein complex’s stability as well as the identification of conformational changes caused by ligand binding.

All docked complexes were improved and evaluated using GROMACS 2021.3 with the CHARMM36 force field^56^. TIP3P water molecules were used to dissolve the protein-ligand complexes in a dodecahedron box^57^, with a minimum spacing of 1.0 nm between the protein and box edges. Na^+^ and Cl^–^ ions were used to neutralize the system at a physiological concentration of 150 mM. Energy minimization was carried out using the steepest descent technique until the maximal force was less than 1000 kJ/mol/nm. The systems were then equilibrated using an NVT ensemble for 500 ns at 300 K (controlled by the V-rescale thermostat), then an NPT ensemble for 500 ns at 300 K (controlled by the Nosé-Hoover thermostat)^58^ and 1 atm (controlled by the Parrinello-Rahman barostat)^59^. The MD simulations were performed on the Alexandria Library Supercomputer’s compute nodes, which were each equipped with two Intel Xeon Gold 6248 processors and 192 GB of RAM, with a total simulation time of 500 ns per ligand and a time step of 2 fs. Long-range electrostatics were modeled using periodic boundary conditions and the Particle Mesh Ewald (PME) technique^60^. LINCS restrictions were imposed to all hydrogen-containing bonds^61^.

The resulting molecular dynamics (MD) trajectories were analyzed using GROMACS tools^51^ (g_rms, g_rmsf, g_hbond, g_mindist) to calculate the root-mean-square deviation (RMSD) for stability, root-mean-square fluctuation (RMSF) of binding site residues, hydrogen bond interactions, and minimum distances between key ligand and protein atoms throughout the simulation, respectively. Additionally, VMD 1.9.3^62^ was employed to visualize the trajectories and generate representative snapshots of the binding modes. Binding free energies for the top-scoring poses of each ligand were computed using the gmx_MMPBSA tool^63^, enabling a comparison of binding affinity and stability relative to K36. To identify critical residues involved in ligand binding, the binding free energy was decomposed into per-residue contributions. High-resolution visualizations were created using BIOVIA Discovery Studio Visualizer v24.1.0.23298^64^ and QtGrace^65^.

## Results and discusions

### Docking studies

**Fig. 2:**
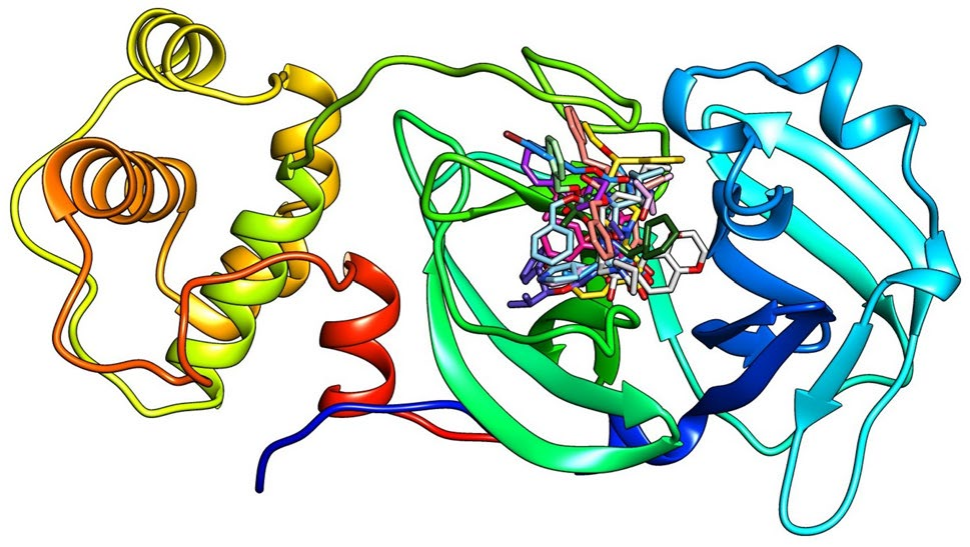
The structural model of the largest active site of the main protease (Mpro) reveals that all investigated inhibitors bind to the same binding pocket as the parent K36 inhibitor. This consistent binding site suggests a shared mechanism of interaction among the inhibitors.

Based on initial cavity detection analysis, the docking process demonstrated that Mpro has more than one active site. The largest pocket (where K36 binds) is composed from twenty (20) amino acids (namely: Thr25, Thr26, Leu27, His41, Val42, Ser46, Met49, Pr052, Tyr54, Phe140, Leu141, Asn142, Gly143, Ser144, Cys145, His163, His164, Met165, Glu166, His172). Each amino acid of this group has its crucial role in forming the active site cavity to attract the inhibitor. From inspection of Figure 2, all the explored inhibitors are found to be located in the same pocket, and bound to the same amino acids (His41 and Cys145 from Mpro). This confirms the ability of the given inhibitors to interact in a similar way as K36, and hence resulting in disease prevention.

The docking scores (DS) of the selected ligands (KL1 to KL10) in complex with the main protease (Mpro) are presented in Table 2. The docking score, which serves as a computational indicator of binding affinity, demonstrates that all proposed ligands exhibit comparable DS values to the parent compound K36. Among the ligands, KL1 exhibits the lowest DS value (-10.23 kcal/mol), while KL7 shows the highest DS value (-13.45 kcal/mol), compared to -11.87 kcal/mol for K36. This variation in docking scores can be attributed to the enhanced reactivity of the proposed ligands, as illustrated by their chemical structures in Figure 1.

As shown in Table 2, all K36 analogues (KL1-KL10) exhibited superior docking scores compared to clinically investigated inhibitors. Notably, KL7 achieved the most favorable DS (-13.54 kcal/mol), outperforming both the parent compound K36 (-11.87 kcal/mol) and FDA-approved drugs like nirmatrelvir (-8.7 kcal/mol). This consistent enhancement suggests our structural modifications successfully improved binding affinity relative to existing scaffolds.

**Table 2:** Docking Score (DS) Performance for each ligand with Mpro. Data obtained from docking process using MOE 2009 software. Values are in kcal/mol. Docking scores for literature-reported Mpro inhibitors have been given for comparison.

Present study		Previous studies	
System	Docking score	System	Docking score
Mpro-K36	-11.87	Nirmatrelvir^66^	-8.7
Mpro-KL1	-10.23	Vorapaxar^67^	-8.9
Mpro-KL2	-11.54	Boceprevir^68^	-6.8
Mpro-KL3	-11.54	Alpha-Ketoamide 13b^7^	-8.2
Mpro-KL4	-11.83	FB2001^69^	-8.9
Mpro-KL5	-11.54	PBI-0451^70^	-9.2
Mpro-KL6	-12.45	Ensitrelvir^71^	-11.2
Mpro-KL7	-13.54	Lopinavir^72^	-6.2
Mpro-KL8	-10.76	Dutasteride^67^	-9.9
Mpro-KL9	-11.23	Ergotamine^67^	-9.8
Mpro-KL10	-10.41	d-Tubocurarine^67^	-9.5

**Fig. 3:**
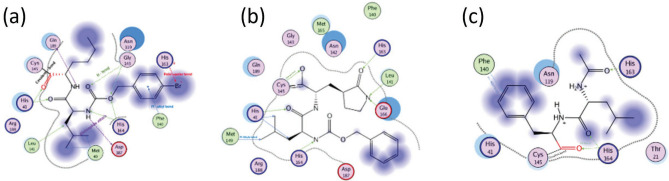
Different molecular interactions, including covalent, hydrogen, and $\pi$-alkyl bonds, of the main protease (Mpro) with three selected ligands exhibiting varying affinities: KL7 (high affinity, panel a), K36 (parent inhibitor, panel b), and KL2 (low affinity, panel c). In each panel, the inhibitor number is indicated, and the interacting amino acid residues are highlighted along with their specific interactions.

Figure 3 illustrates the molecular interactions of the main protease (Mpro) with three selected ligands exhibiting varying affinities: KL7 (high affinity, panel a), K36 (parent inhibitor, panel b), and KL2 (low affinity, panel c). In panel a, KL7 demonstrates significant interactions with the Mpro active site, supported by strong electron density maps, indicative of its potent inhibitory effect. KL7 (high affinity) demonstrates superior binding through three stable hydrogen bonds with Gln189 (2.8 Å), Gly143 (3.0 Å), Asn142 (2.9 Å), and His164 (3.1 Å), complemented by a $\pi$-alkyl interaction with Met49 (4.1 Å) and extensive hydrophobic contacts with Met165 (91% occupancy) and Pro168 (87% occupancy). In contrast, K36, the parent inhibitor, exhibits a less extensive interaction network compared to KL7, consistent with its lower binding affinity. This parent inhibitor shows moderate binding via covalent anchoring to Cys145 (85% occupancy), hydrogen bonds with Gln189 (3.0 Å), His164 (2.9 Å), and Asn142 (3.1 Å), along with $\pi$-alkyl interactions with Met49 (4.3 Å) and Leu141 (4.5 Å). Panel c reveals that KL2, the weakest binder, displays sparse electron density and fewer contacts, reflecting its low affinity. In contrast, KL2 (low affinity) exhibits weaker binding characterized by intermittent hydrogen bonds with His163 (3.3 Å), His164 (3.2 Å), and Phe140 (3.4 Å), reduced hydrophobic contacts (Met49: 38% occupancy), and higher structural fluctuations during simulations. This comparative analysis highlights KL7’s optimal combination of polar and hydrophobic interactions, K36’s covalent anchoring strategy, and KL2’s suboptimal contact network, providing valuable insights for structure-based inhibitor design targeting Mpro.

### Stability and dynamic conformations of protein-ligand complexes

The stability of protein-ligand complexes is influenced by solvent conditions and molecular interactions. It has been observed in several cases that compounds with favorable docking scores and strong molecular interactions may fail to bind to the protein in experimental settings. To address this, the structural behavior, dynamics, and flexibility of the ten proposed ligand complexes were evaluated over a 500 ns molecular dynamics (MD) simulation. The interactions between the main protease (Mpro) and the investigated ligands were characterized using hydrogen bond analysis, root-mean-square deviation (RMSD), root-mean-square fluctuation (RMSF), radius of gyration (Rg), and binding free energy calculations. This comprehensive analysis provides a detailed assessment of the binding stability, dynamic interactions, and inhibitory potential of the analogues. By evaluating these parameters, we aim to identify promising candidates for further development as antiviral agents against COVID-19.

**Fig. 4:**
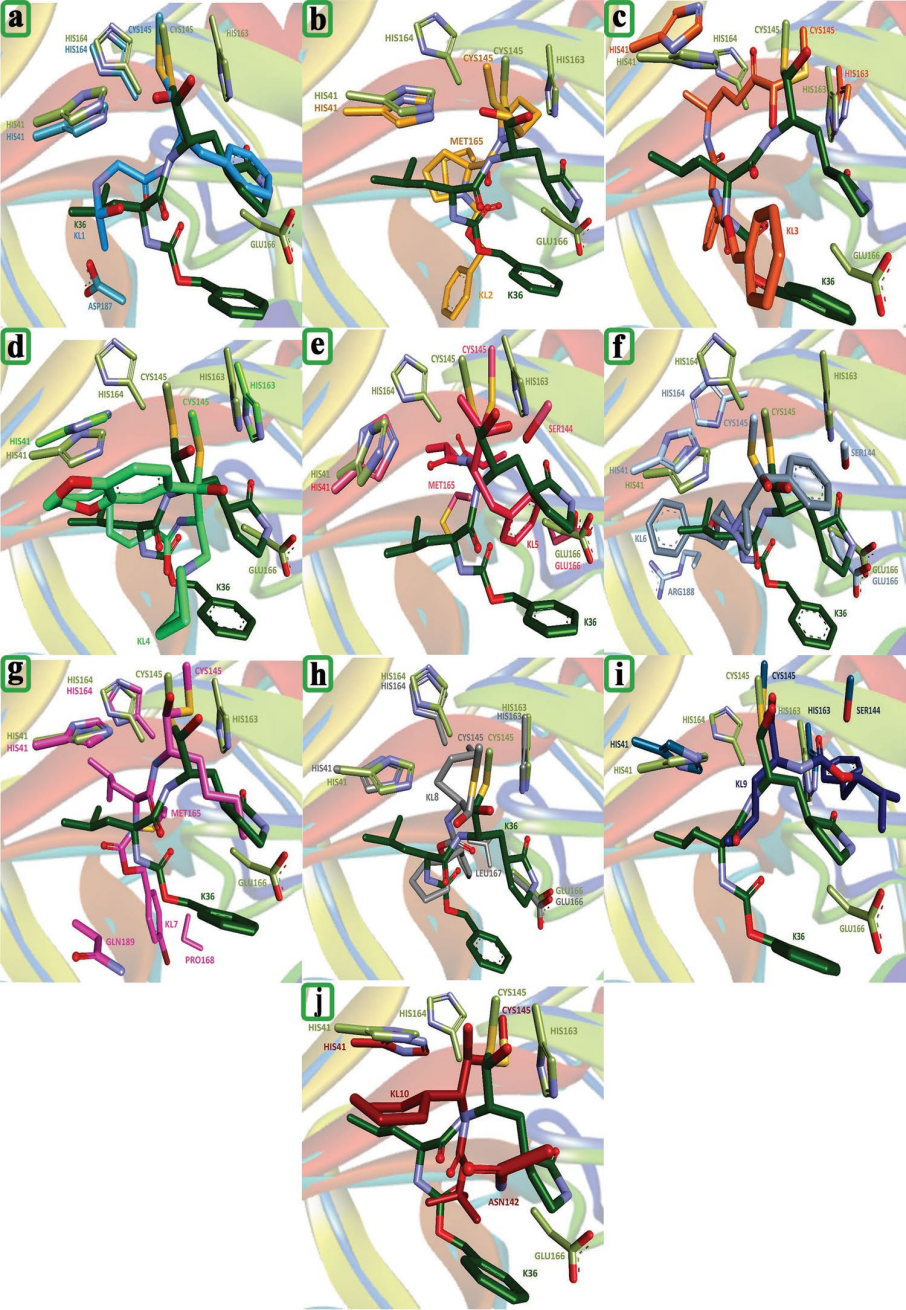
The overlay of the Mpro-K36 complex (green) with Mpro complexes bound to other ligands (KL1 to KL10, shown in various colors). The overlay highlights key amino acid interactions and structural features that contribute to the effective inhibition of Mpro.

The overlay procedure involves superimposing the three-dimensional (3D) structures of Mpro complexed with various ligands, allowing for a direct comparison of their binding mechanisms and interactions within the Mpro active site, as illustrated in Figure 4. This approach aims to identify critical amino acid interactions and structural features that contribute to the effective inhibition of Mpro. KL1, KL2, and KL3 exhibit binding mechanisms similar to K36, forming strong interactions with Cys145, His41, and Glu166. In contrast, KL4, KL5, KL6, and KL7 show greater divergence from K36’s binding conformation, potentially engaging additional amino acids such as Ser144, Met165, and Gln189. Similarly, KL8 and KL9 deviate from K36’s binding mechanism, with possible interactions involving Leu167 and Arg188. KL10 demonstrates the most significant divergence from K36’s binding mode, with potential interactions including Asn142 and Asp187.

To comprehensively investigate the ligand$\cdots$Mpro interactions, several parameters derived from molecular dynamics (MD) calculations will be analyzed in detail in the following subsections.

#### Hydrogen bond analysis

**Fig. 5:**
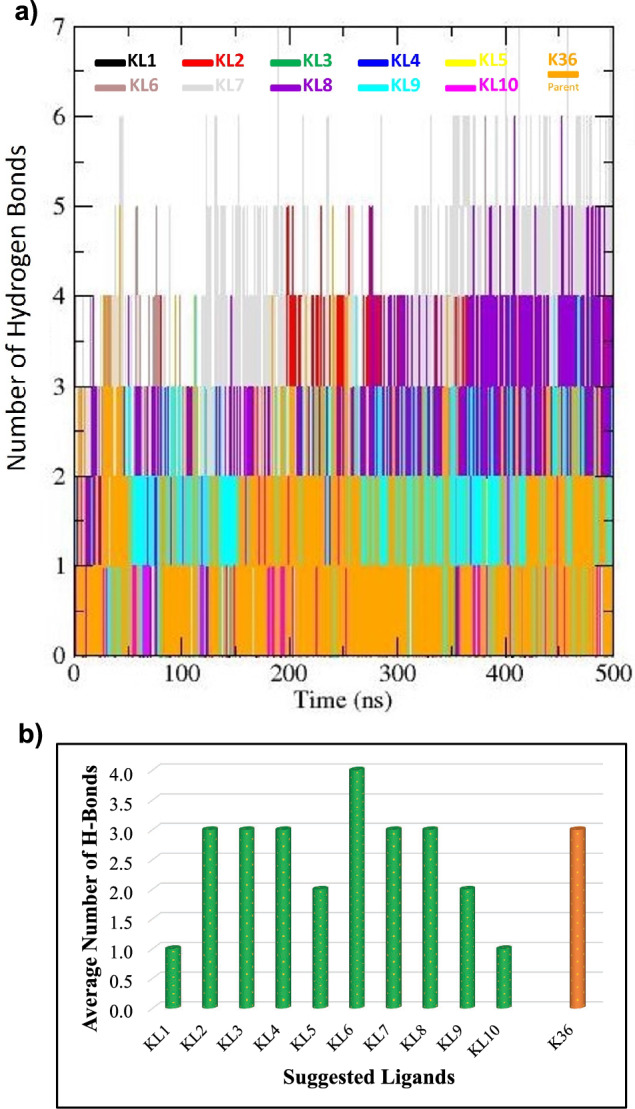
The number of hydrogen bonds formed between the main protease (Mpro) and various ligands (KL1 to KL10). Panel (a) illustrates the fluctuation in the number of hydrogen bonds over a 500 ns simulation, with different colored stacked bars representing each inhibitor. Panel (b) displays the average number of hydrogen bonds, compared to the parent inhibitor, K36.

Hydrogen bonds play a critical role in ligand binding and recognition. In this study, we focused on analyzing the hydrogen bonding interactions between ten potential inhibitors (KL1 to KL10) and the main protease (Mpro), in comparison to the parent inhibitor K36 (see Figure 5). The number of hydrogen bonds formed between each ligand and Mpro exhibited significant variation over a 500 ns simulation, highlighting the dynamic nature of these interactions (Figure 5, panel a). This variability suggests that the ligands interact with the protein in distinct ways, likely due to differences in their chemical structures and the specific amino acid residues they engage within the protein’s binding site. A higher capacity for hydrogen bonding interactions was demonstrated by the fact that certain ligands consistently formed more hydrogen bonds than others. Notably, KL6 consistently established the highest number of hydrogen bonds (approximately 4) (Figure 5, panel b), indicating it possesses a greater quantity of hydrogen bond donors and acceptors compared to other ligands.

The amino acids Cys145 and His41 in the Mpro active site are critical for inhibitor binding. Almost ligands investigated in this study, including K36, contain a carbonyl group, and hence are capable of forming a covalent bond with Cys145, a key feature often associated with Mpro inhibition. The formation of this covalent bond is frequently a crucial element in the inhibition of Mpro. Additionally, the ligands feature functional groups that can interact with His41 through hydrogen bonding or other polar interactions. Ligands with a higher number of hydrogen bond donors and acceptors, such as hydroxyl (-OH) and amine (-NH_2_) groups, are more likely to form a greater number of hydrogen bonds with the protein. Furthermore, the spatial arrangement of these functional groups within the ligand molecule can significantly influence the hydrogen bonding interactions.

From a chemical perspective, KL1, KL2, KL3, and K36 contain carbonyl (C=O) groups (see Figure 1). These functional groups act as hydrogen bond acceptors for the thiol (-SH) group of Cys145. Additionally, these ligands feature nitrogen atoms capable of interacting with the imidazole ring of His41. The KL4 ligand includes an amide group (-NH_2_), which can function as both a hydrogen bond donor and acceptor, enabling interactions with both Cys145 and His41. In contrast, KL5, KL6, KL7, KL8, KL9, and KL10 exhibit a reduced capacity for hydrogen bonding interactions with Cys145 and His41 compared to the other ligands. These ligands may rely more heavily on hydrophobic interactions or alternative polar interactions with other amino acids in the binding site.

#### Root mean squared deviation (RMSD)

**Fig. 6:**
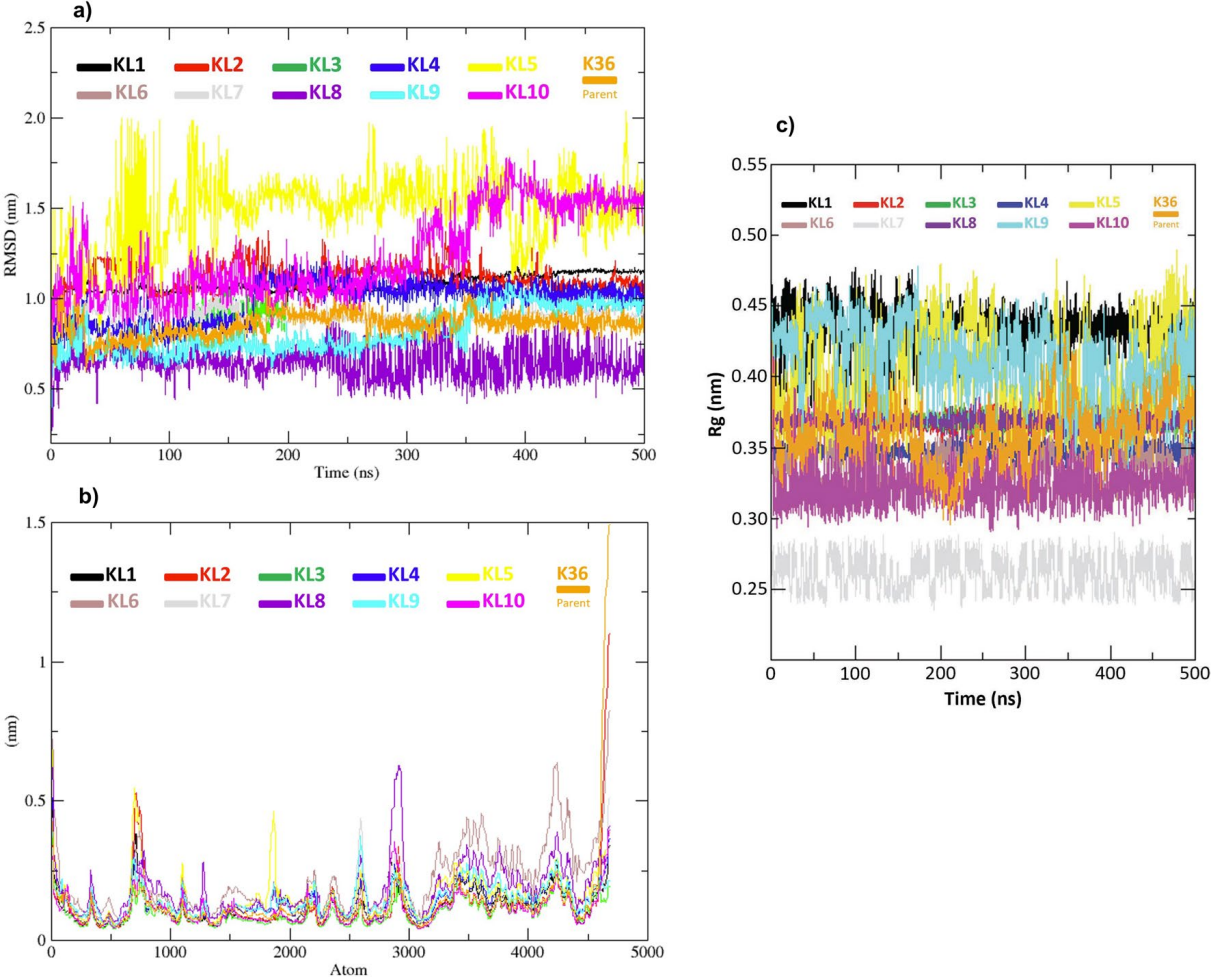
Molecular dynamics simulation: a) RMSD, b) RMSF, and c) Rg for the suggested ten ligand-protein complexes, including the parent inhibitor K36. Data were derived from 500 ns MD simulations.

RMSD is a key metric used to measure the structural stability of a ligand-protein complex over time. It quantifies the average deviation of atomic positions from a reference structure, providing insights into the stability and consistency of binding interactions. In this study, the RMSD analysis revealed that most inhibitors exhibited stable binding to the main protease (Mpro), with RMSD values ranging from 0.5 to 2 nm, see Figure6, panel a. This indicates that these inhibitors maintained consistent interactions with the main protease throughout the 500 ns simulation. However, certain inhibitors, such as KL5 and KL2, displayed higher RMSD fluctuations, suggesting a more dynamic binding mode. These variations may arise from weaker interactions with the Mpro and the presence of multiple binding conformations.

The inhibitor KL7 emerged as a particularly promising candidate, exhibiting significantly lower RMSD values (0.61 nm) throughout the simulation, as shown in Table 3. This suggests that KL7 maintains a more stable binding orientation and may possess a higher affinity for the main protease compared to other inhibitors. The exceptional stability of KL7 can likely be attributed to specific interactions with the protease active site, such as hydrogen bonds, hydrophobic contacts, or $\pi$-$\pi$ stacking. Additionally, the presence of a bromine atom on the hexagonal ring (Figure 1) contributes to reduced molecular motion, thereby minimizing fluctuations. In contrast, KL5 and KL2 displayed the highest RMSD values (1.53 and 1.15, respectively), indicating greater fluctuations and reduced binding stability. These variations may result from weaker interactions and less favorable binding conformations with the protease. It is worth noting that the average RMSD values for all ligands are relatively close to that of the parent inhibitor K36, suggesting their potential to serve as viable alternatives to the experimental inhibitor K36.

The observed RMSD patterns provide valuable insights into the dynamics of protease-inhibitor interactions, guiding the development of more effective inhibitors. For instance, the stability of KL7 suggests that its binding interactions could serve as a model for designing new inhibitors with enhanced binding affinities. Conversely, the fluctuations observed for KL5 and KL2 indicate that structural modifications may be necessary to improve their binding stability.

**Table 3:** Average Root Mean Square Deviation (RMSD) values (in nm) for the parent inhibitor K36 and its ten analogues (KL1–KL10), derived from molecular dynamics simulations.

Inhibitor	K36	KL1	KL2	KL3	KL4	KL5	KL6	KL7	KL8	KL9	KL10
Average RMSD (nm)	0.86	0.81	1.15	0.81	0.83	1.53	0.86	0.61	0.96	0.81	1.01

#### Root mean squared fluctuation (RMSF)

Root Mean Square Fluctuation (RMSF) quantifies molecular flexibility by measuring the average displacement of atoms from their mean positions during simulations. This analysis identifies regions of high and low mobility within biomolecules, providing crucial insights into functional dynamics such as active site flexibility and ligand-induced conformational changes. The role of flexibility is complex: while moderate loop mobility facilitates ligand entry and induced-fit binding, excessive fluctuations near binding sites can destabilize protein-ligand complexes. Effective therapeutic candidates typically stabilize the protein structure, particularly in critical regions like active sites, while maintaining necessary functional motions. This balance between stability and flexibility is essential for optimal ligand performance.

The data presented in Figure 6, panel b corresponds to an RMSF analysis conducted on the main protease (Mpro) complexed with the reference molecule K36 and its ten analogues. These simulations spanned 500 ns, providing sufficient time to assess the dynamic behavior of the protein-ligand complexes. A closer examination of the RMSF data for K36 reveals an average fluctuation of 0.32 nm, with values ranging from 0.09 to 1.5 nm. This distribution indicates that certain regions of the protein exhibit greater flexibility than others. The largest fluctuations are observed in loop regions and the C-terminal domain, as these areas are typically more dynamic and less structured compared to the rest of the protein. In contrast, the ten analogues exhibited lower RMSF values than K36, with average fluctuations ranging between 0.11 and 0.21 nm. This suggests that the analogues bind more tightly to the protein, reducing the flexibility of its backbone. The enhanced stability observed with the analogues may be attributed to stronger interactions with the protein, leading to a more stable complex.

A particularly noteworthy finding is that all analogues exhibit minimal variability in the active site region. This observation suggests that the ligands effectively stabilize this critical area of the protein, potentially disrupting its function and leading to inhibition. This result underscores the potential of these analogues as potent inhibitors of the main protease.

The fluctuations observed in the RMSF analysis can be attributed to various chemical properties of the ligand structures. Smaller, more rigid molecules, such as KL1, KL2, and KL10, tend to restrict the protein’s mobility, resulting in lower RMSF values. In contrast, molecules with multiple hydrogen bond donors and acceptors, such as K36, KL4, and KL5, can form strong interactions with the protein, potentially increasing RMSF values in specific regions due to localized flexibility. Hydrophobic groups in ligands, such as those in KL3, KL7, and KL9, enhance overall stability and reduce RMSF values by interacting with nonpolar residues. Specific observations, such as the pronounced RMSF peaks of KL2 and K36, can be explained by their distinct structural features.

#### Radius of gyration (Rg) and compactness

This section presents an analysis of the Radius of Gyration (Rg) values for the ten ligands (KL1 to KL10) and the parent inhibitor K36 in complex with the main protease, as shown in Figure 6, panel c. The Rg values, derived from 500 ns molecular dynamics simulations, serve as an indicator of molecular compactness. Variations in Rg over time for each ligand reflect differing levels of flexibility in the protein-ligand complexes.

Examining each ligand individually reveals the following observations: KL1 exhibits significant variations in Rg, ranging between 0.42 and 0.52 nm, indicating a dynamic and less compact complex. KL2 demonstrates the most stable Rg values, consistently ranging from 0.28 to 0.34 nm, suggesting a highly compact and rigid complex. KL3 shows moderate variations in Rg, oscillating between 0.30 and 0.50 nm, reflecting a balance between compactness and flexibility. KL4 displays Rg values consistently ranging from 0.38 to 0.42 nm, indicating a relatively stable and compact complex. KL5 exhibits the widest range of Rg values, spanning from 0.30 to 0.60 nm, suggesting a highly flexible complex with varying degrees of compactness. KL6 has Rg values between 0.40 and 0.44 nm, indicating a stable complex with moderate variability. KL7 exhibits behavior similar to KL6, with Rg values ranging from 0.29 to 0.40 nm, reflecting a stable complex with minimal fluctuations. KL8 shows Rg values comparable to KL2, consistently ranging from 0.28 to 0.34 nm, indicating a highly compact and rigid complex. KL9 shows moderate variations in Rg, spanning 0.40 to 0.50 nm, similar to KL3, reflecting a balance between compactness and flexibility. KL10 exhibits Rg values between 0.34 and 0.46 nm, indicating a reasonably stable complex with minor variations. The parent inhibitor K36 demonstrates Rg values similar to KL6 and KL7, ranging from 0.39 to 0.43 nm, indicating a stable complex with moderate variability.

These findings highlight the diverse effects of the ligands on the structural dynamics of the main protease. Ligands such as KL2 and KL8 form highly compact complexes, while ligands like KL1 and KL5 result in more flexible complexes. The average Rg values vary among the ligands, likely reflecting their distinct impacts on the protein’s stability and dynamics, which are critical factors in drug design and protein engineering.

#### Molecular mechanics poisson-boltzmann surface area (MM-PBSA) binding energy

MM-PBSA (Molecular Mechanics Poisson-Boltzmann Surface Area) is a computational method used to estimate the binding free energy of protein-ligand complexes. It combines molecular mechanics (MM) with continuum solvation models (Poisson-Boltzmann and Surface Area) to calculate the energy contributions that determine how strongly a ligand binds to a protein.

In a series of protein-ligand complexes involving the primary ligand (K36) and its analogues (KL1–KL10), a comprehensive analysis of binding energies and contributing factors was conducted, as summarized in Table 4. The binding energy analysis reveals that KL7 and KL6 are the most promising inhibitors, with binding energies of -34.57 kJ/mol and -31.45 kJ/mol, respectively. KL7 outperforms the parent inhibitor K36 (-20.029 kJ/mol), primarily due to its strong van der Waals interactions (-58.542 kJ/mol) and favorable hydrophobic contributions (SASA energy: -7.025 kJ/mol). In contrast, KL1 and KL10 exhibit weaker binding energies (-17.478 kJ/mol and -18.556 kJ/mol, respectively), consistent with their lower van der Waals and electrostatic interactions. The data highlight the dominant role of van der Waals forces in stabilizing the protein-ligand complexes, as evidenced by the strong correlation between van der Waals energy and binding affinity. Electrostatic interactions, while contributing to binding, play a secondary role, as seen in K36, which has strong electrostatic energy (-13.54 kJ/mol) but only moderate binding affinity due to weaker van der Waals contributions.

Polar solvation energy, representing the desolvation penalty, is highest for K36 (53.475 kJ/mol), partially offsetting its favorable electrostatic interactions. Ligands like KL10, with lower polar solvation energy (32.004 kJ/mol), still exhibit weak binding due to insufficient van der Waals and electrostatic contributions. The SASA energy, reflecting hydrophobic interactions, further underscores the importance of nonpolar interactions in binding stability. For instance, KL7’s strong SASA energy (-7.025 kJ/mol) complements its van der Waals interactions, enhancing its overall binding affinity. These findings suggest that optimizing van der Waals and hydrophobic interactions, while balancing electrostatic and desolvation effects, is key to designing more potent and selective inhibitors.

**Table 4:** Van der Waals energy, electrostatic energy, polar solvation energy, solvent-accessible surface area (SASA) energy, and binding energy (in kJ/mol ) for all studied systems.

System	van der Waals	Electrostatic	Polar solvation	SASA	Binding
Mpro-K36	-53.09	-13.53	53.47	-6.80	-20.03 ± 1.96
Mpro-KL1	-40.00	-9.10	36.43	-4.80	-17.48 ± 1.95
Mpro-KL2	-46.20	-10.21	44.28	-5.54	-21.45 ± 2.37
Mpro-KL3	-56.11	-11.12	44.48	-6.74	-29.49 ± 2.93
Mpro-KL4	-54.02	-12.11	51.25	-6.48	-21.36 ± 2.22
Mpro-KL5	-42.12	-9.45	37.82	-4.69	-18.44 ± 1.95
Mpro-KL6	-59.00	-7.99	39.54	-4.00	-31.44 ± 3.19
Mpro-KL7	-58.54	-10.33	41.32	-7.02	-34.57 ± 3.24
Mpro-KL8	-49.89	-9.88	39.52	-5.98	-26.23 ± 2.74
Mpro-KL9	-45.85	-9.21	33.55	-5.50	-27.01 ± 2.81
Mpro-KL10	-38.01	-8.07	32.08	-4.55	-18.55 ± 1.93
Nirmatrelvir^66^	–	–	–	–	-12.5 ± 1.2
Ensitrelvir^71^	–	–	–	–	-11.2 ± 0.9
PBI-0451^70^	–	–	–	–	-10.3 ± 1
Alpha-Ketoamide 13b^7^	–	–	–	–	-9.8 ± 0.8

The results identified KL7 as the most effective binder, outperforming even the parent ligand K36, with a binding energy of -34.57 kJ/mol. Notably, KL7 exhibits significantly enhanced binding energy compared to previously reported inhibitors in the literature, as demonstrated in Table 4.

The binding energies in this dataset range from -34.57 to -17.48 kJ/mol, with a mean of -26.03 kJ/mol and a standard deviation of 8.54 kJ/mol. This moderate variability suggests that the structural modifications introduced in the ligand analogues significantly influence their binding affinity to the protein target. The absence of outliers indicates that all data points fall within an acceptable range and likely represent genuine binding interactions.

The main reason behind the high stability of KL7 inhibitor is its structural features (e.g., the moiety containing the bromine atom, its overall shape and functional groups shown in Figure 1). Additionally, we link its superior MM-PBSA binding energy (-34.57 kJ/mol) to favourable energy components (specifically mentioning its strong van der Waals energy: -58.54 kJ/mol, and favourable SASA energy: -7.03 kJ/mol from Table 4). We correlate this with its observed stability in MD (lowest average RMSD [0.61 nm] from Table 3 and Figure 6 a, suggesting a stable binding conformation. We connect these observations back to specific interactions, noting that while KL6 showed the highest average H-bond count (Figure 5 b), KL7’s combination of H-bonds (with key residues like His41/Cys145), strong hydrophobic/van der Waals contacts (facilitated by its structure), and conformational rigidity (possibly enhanced by the bromine substituent leading to low RMSD/Rg) results in the most favourable overall binding free energy according to the MM-PBSA calculation. Understanding the molecular basis of its enhanced binding affinity, particularly the dominant role of van der Waals interactions, can provide valuable insights for designing more potent and selective inhibitors.

### Reproducibility and validation via replica simulations

To ensure the robustness and reproducibility of our findings on the parent inhibitor K36 and its analogues (KL1–KL10) with the main protease (Mpro), we employed a rigorous methodology that involved repeating each simulation. Given the sensitivity of molecular dynamics (MD) simulations to initial conditions, we conducted each simulation three times (simulations 1, 2, and 3), each of which began with a distinct set of randomly generated velocities. This approach aimed to minimize the influence of initial conditions on the observed trajectories and provide a more comprehensive understanding of the system’s dynamic behavior.

**Fig. 7:**
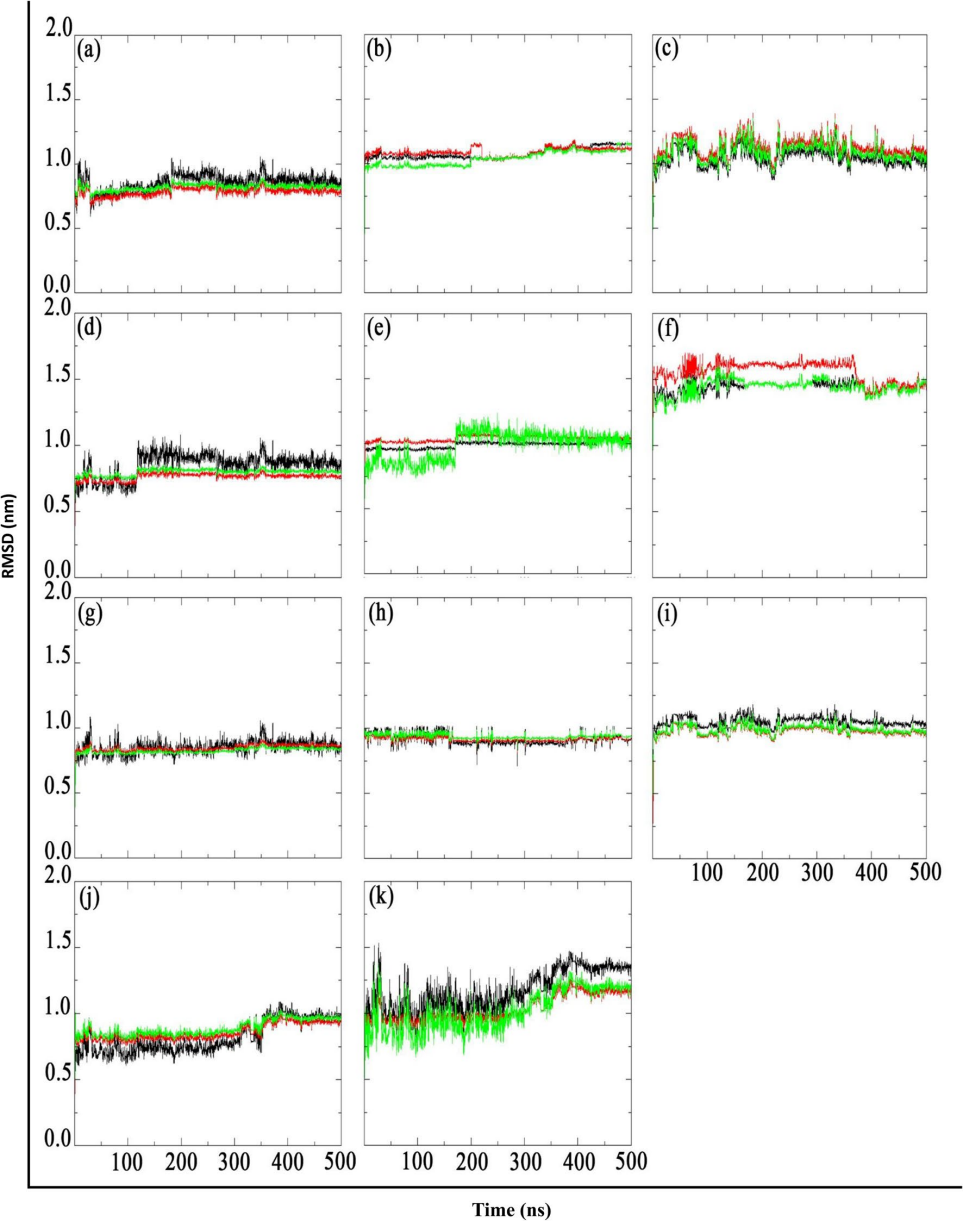
Root mean square deviation (RMSD) of K36 and its 10 analogues after least-squares fitting to the backbone. The black, red, and green lines represent simulations 1, 2, and 3, respectively, for each inhibitor.

To validate the consistency of our results, we monitored key parameters, such as root mean square deviation (RMSD), in multiple replicates (see Figure 7). As shown in the figure, the RMSD trajectories for each system exhibit high consistency across replicates, with minimal deviations between runs. For example, the RMSD values for all systems stabilize within a narrow range (e.g. 0.5 to 2.0 nm) after an initial equilibration phase, demonstrating the reliability of our simulations. This strategy not only enhanced the reliability of our findings but also allowed us to identify any discrepancies arising from the stochastic nature of the simulations.

Significant variations between replicates, if observed, would necessitate further investigation, potentially requiring longer simulation times or improved sampling techniques to achieve reliable results. By conducting multiple simulations: simulation1, 2, and 3 (black, red, and green trajectories within Figure 7), we ensured the validity of our conclusions and advanced a more robust understanding of the system under study. It worth noting that, the standard deviation of the average RMSD across the three replicates was typically below 0.15 nm for most systems after equilibration, confirming high consistency between independent runs.

## Conclusion

This study performed a comprehensive computational analysis to evaluate ten K36-based compounds (KL1-KL10), derived from the parent inhibitor K36, as potential inhibitors of the main protease SARS-CoV-2 (Mpro). Using molecular docking, molecular dynamics (MD) simulations, and binding free energy calculations, we systematically investigated the interactions between these compounds and Mpro. KL7 emerged as the most promising candidate, exhibiting the strongest binding affinity (docking score: -13.54) and exceptional stability (RMSD: 0.61 nm), attributed to its interactions with key residues such as Cys145 and His41, including hydrogen bonds, hydrophobic contacts, and $\pi$-$\pi$ stacking. The presence of a bromine atom in the KL7 structure further contributed to its reduced fluctuations and enhanced stability.

Hydrogen bond analysis revealed that KL6 formed the highest number of hydrogen bonds ( 4), indicating a superior capacity for hydrogen bonding interactions. Radius of gyration (Rg) analysis demonstrated varying levels of compactness and flexibility among the Mpro-ligand complexes, ligands such as KL2 and KL8 forming highly compact structures, while KL1 and KL5 exhibited greater flexibility. Binding free energy calculations using the gmx_MMPBSA program confirmed KL7 as the most effective binder (-34.57 kJ/mol, MM-PBSA binding energy), outperforming the parent inhibitor K36. A strong positive correlation between the binding energy and van der Waals interactions highlighted the importance of these forces in stabilizing the complexes.

In conclusion, this study identified several promising K36 analogues as potential inhibitors of the main protease of SARS-CoV-2, KL7 being a leading candidate due to its high binding affinity, exceptional stability, and favorable interactions with critical residues. These findings provide valuable insights for the design of potent antiviral drugs targeting COVID-19, paving the way for further experimental validation and clinical development. While these computational results highlight KL7 as a promising candidate, experimental studies are needed to confirm its inhibitory activity, pharmacokinetic properties, and safety profile in vitro and in vivo.

## Data Availability

The datasets used and/or analyzed during the current study are available from the corresponding author on reasonable request.

## Abbreviations

ACE2: Angiotensin-Converting Enzyme 2
COVID-19: Coronavirus Disease 2019
DB: Drug Bank
DS: Docking Score
FMO: Fragment Molecular Orbital
GC376: A covalent inhibitor of SARS-CoV-2 Mpro
GROMACS: GROningen MAchine for Chemical Simulations
KL1-KL10: K36-based inhibitor analogs
K36: A covalent inhibitor of SARS-CoV-2 Mpro
MD: Molecular Dynamics
MM-PBSA: Molecular Mechanics Poisson-Boltzmann Surface Area
MOE: Molecular Operating Environment
Mpro: Main Protease (also known as 3CLpro)
NSPs: Non-Structural Proteins
PDB: Protein Data Bank
PME: Particle Mesh Ewald
RMSD: Root Mean Square Deviation
RMSF: Root Mean Square Fluctuation
Rg: Radius of Gyration
SARS-CoV-2: Severe Acute Respiratory Syndrome Coronavirus 2
SASA: Solvent-Accessible Surface Area
VMD: Visual Molecular Dynamics
